# Two parallel chromosome‐level reference genomes to support restoration and aquaculture of European flat oyster *Ostrea edulis*

**DOI:** 10.1111/eva.13465

**Published:** 2022-08-16

**Authors:** Tim P. Bean, Arnaud Tanguy, Carolina Peñaloza, Manu Kumar Gundappa, Isabelle Boutet, Ross D. Houston, Daniel J. Macqueen, Pierre Boudry

**Affiliations:** ^1^ The Roslin Institute and Royal (Dick) School of Veterinary Studies University of Edinburgh, Easter Bush Campus Edinburgh UK; ^2^ CNRS, UMR 7144, Station Biologique de Roscoff Sorbonne Université Roscoff France; ^3^ Département Ressources Biologiques et Environnement Ifremer Plouzané France

**Keywords:** aquaculture, bivalve, genomics, mollusc, restoration, shellfish

## Abstract

This volume of Evolutionary Applications sees the publication of two genomes for the European native flat oyster *Ostrea edulis*, a species of significant evolutionary, ecological and commercial value. Each is a highly contiguous chromosome‐level assembly from individuals of different genetic backgrounds, which have been benchmarked against one another. This situation has resulted from the serendipitous discovery that two independent research groups were both deep into the process of building, annotating and investigating separately produced assemblies. Due to constraints with funder requirements and the need to recognize early career researchers for their work, alongside the technical challenge of integrating assemblies from two very different genomes, there was limited capacity to merge the sequences into one publication at the stage of discovery. This issue is likely to become very common over the next few years until the technologies for working with multiple genomes at once, for example, graph genomes, become commonplace in nonmodel species. Consequently, both of our teams have decided to collaborate rather than compete, recognizing the benefit to copublishing two separate genome resources for the research community, each with distinct scientific investigations, and working collaboratively to benchmark the assemblies.

This volume of Evolutionary Applications includes the deliberate simultaneous publication of two highly contiguous, chromosome‐level reference genome assemblies for the native European flat oyster *Ostrea edulis* (Boutet et al., 2022; Gundappa et al., 2022). *Ostrea edulis* is an iconic native species across Europe where it was heavily fished and consumed through to the early 1900s. Natural beds extend in shallow waters from the Norwegian coast of North Sea, along the Atlantic Ocean down to the Mediterranean and through to the Black Sea. Non‐native *O. edulis* populations have naturalized in Atlantic waters of North America, namely in Nova Scotia, New Brunswick and Maine. The commercial success of this species in Europe and heavy mortality resulting from two parasitic diseases eventually resulted in widespread collapse of fisheries, and stocks have remained in a depleted state ever since (Laing et al., 2006). Scientific interest in this species has, however, recently seen a resurgence due to burgeoning activities associated with ecological restoration (Pogoda et al., 2019; Zu Ermgassen et al., 2020), and a revival in aquaculture production in Europe, as a complement to the Pacific oyster *Crassostrea gigas*. As such, it is expected that the timing of these genome releases will provide an extremely useful foundation for the purposes of comparative genomics (Regan et al., 2021), population genetics (Šegvić‐Bubić et al., 2020), selective breeding (Gutierrez et al., 2018; Naciri‐Graven et al., 1998; Peñaloza et al., 2022) and deep explorations into the biology of this species (see Colsoul et al., 2021 for review). Moreover, the joint effort to publish these resources at the same time, in the same journal issue, with overlapping authorship, shows the collaborative nature of those working in this field. However, we also feel there is value in sharing details of the circumstances that led to the publishing of two separate genomes and the scientific potential of having access to multiple genomes for a single species.

Compared with virtually all the classic model species, molluscs are understudied and are fundamentally different from all model taxa on which animal genomic techniques were pioneered (Hedgecock et al., 2005). This is perhaps best highlighted amongst the bivalve molluscs, where several key observations exemplify extreme genetic complexity. Primarily, bivalves exhibit exceptionally high levels of polymorphism and genomic plasticity, which appears to persist even with high levels of consanguineous mating (Zhang et al., 2012). This variation has been observed from the level of marker heterozygosity (Hedgecock et al., 2005) through to significant structural variation (Jiao et al., 2021) including measures of interindividual genomic divergence of 0.21 (calculated by dividing the total length of the divergent regions by genome size, Qi et al., 2021), and gene presence–absence variation suggesting one‐third of genes in a mussel pan‐genome (includes core genes essential for all individuals, and dispensable genes found only in some) are dispensable (Gerdol et al., 2020). This high level of variability results in high heterozygosity, including in the *Ostrea* genome published in this volume of Evolutionary Applications, which has a heterozygosity rate of 1.07% (Gundappa et al., 2022). As such, the assembly of high‐quality reference genomes for bivalve species has historically proved challenging (Davison & Neiman, 2021), in part due to the difficulty in identifying or breeding inbred animals (Dégremont et al., 2022; Zhang et al., 2012).

An associated peculiar observation is that wild cohorts of oyster are often observed to have a low effective population size relative to census population sizes (e.g. Ne/N of lower than 10^−5^ in *C. gigas* but also low ratios in *O. edulis*) (Hedgecock, 1994; Lallias et al., 2010), leading to ‘genetic patchiness’ in nature. This is most likely caused by a phenomenon known as a ‘sweepstakes reproduction strategy’, which occurs when few parents successfully mate and suggests that a very small proportion of gametes produced by a population are viable in wild situations, either through physical or through genetic barriers to reproduction (Plough, 2016). From a physical perspective, the peculiar characteristics in *O. edulis* include sperm clustered in spermatozeugmata (O'Foighil, 1989; Suquet et al., 2018), consecutive hermaphroditism, where individuals are able to change sex during reproduction (Cole, 1942; Zapata‐Restrepo et al., 2019) and brooding of larvae, are known to influence the genetics and the reproductive dynamics of the species (Lallias et al., 2010). From a genomics perspective, incompatibilities between parent animals caused by aforementioned structural variation (Gerdol et al., 2020) could potentially be a factor. There are further complications; severe segregation distortion in the form of heterozygous or homozygous deficiencies is frequently observed at nuclear markers in progenies of pair crosses (Dégremont et al., 2022; Plough, 2016). Regardless, the interplay between mating success or failure and population level outcomes is hugely important from the perspective of selective breeding and will likely require a great deal of genomic analysis to fully resolve.

Finally, the way through which this assorted diversity accumulates and is maintained in bivalves is not yet fully elucidated, but there are indications this is achieved by multiple mechanisms, often at play during the gametogenesis and larval phases. This encompasses multiple examples of variation being generated de novo, such as transposon activity (Peñaloza et al., 2021; Zhang et al., 2012), and the suggestion of spontaneous replication errors in the meiotic process associated with high fecundity (Plough & Hedgecock, 2016). The result of these mechanisms is an increase in genomic variation and a high genetic load, some of which is expressed temporally and purged through purifying selection during larval development (Plough, 2016). As recent studies have suggested that genetic diversity may be maintained via temporally balancing selection during the larval phase of oyster development (Durland et al., 2021), further studies that combine genomic approaches with classical Mendelian experimental designs will be key to resolve the remaining uncertainty in these systems, and unravel the function they hold in the oyster life cycle (Durland et al., 2022; Hedgecock, 2022).

All these phenomena come together to form highly successful life histories. In total, there are over 20,000 known bivalve species, which together inhabit virtually all known aquatic (both freshwater and marine) ecosystems (Vaughn & Hoellein, 2018). A key to unravelling the genetics underlying these life histories is the development of reference genomes for the key species in these clades. A reference genome is usually a haploid approximation of a genome sequence for a single species. In some animal groups, such as mammals, more than one individual sequence may be merged into one contiguous sequence. For example, the human genome reference (GRCh38) is composed of DNA sequences from 338 individuals and includes sequences of over 100,000 insertions (Wong et al., 2020). The clear benefit of having one, high‐quality reference genome is that of consensus and convenience. However, in species such as oysters, we must consider whether this method is the most appropriate, or even possible. The hugely variable genomes in bivalves, especially the exceptionally high heterozygosity and structural variation, means that a single reference genome may poorly represent the extent and organization of the polymorphism landscape contained in bivalve genomes.

Leading up to the co‐publication of two distinct *O. edulis* reference genomes, we found ourselves in a situation where two distinct research teams were concurrently working towards the same goal, which was realized at an advanced stage of analysis through a mutual network focussed on *O. edulis* restoration (Pogoda et al., 2019). With the continuing increase in accessibility of sequencing resources, we can perhaps assume that this situation is likely to be common. There are, for example, two chromosome‐level genome assemblies of *C. gigas* (Peñaloza et al., 2021; Qi et al., 2021), with at least two more currently being sequenced (*pers comms*). In the case of *O. edulis*, we agreed from that point onwards to work collaboratively to ensure both genomes were co‐published and co‐released, providing a positive endpoint for the scientific community. Collaboration allowed both teams to share sequence data that improved the quality of both reference genomes. In particular, resequencing data from a pair crosses in France allowed the development of a medium‐density SNP linkage map harnessing the expertise of the team in the UK. This linkage map has been used to confirm the orientation of large blocks of sequence in both genomes and provides confidence on the accuracy of both assembled sequences. We also coordinated submission of both genomes to the public archives (NCBI) to occur on the same day, such that neither ‘came first’, which we felt was a fair outcome for the early career researchers that spent a huge amount of effort building and analysing these resources.

Both *O. edulis* reference genomes are now publicly available. The production, presentation and analysis of multiple genomes is not straightforward, and it is common that one simply supersedes the last, but several techniques have been developed to deal with this situation, most notably ‘graph genomes’ that capture variation in multiple rather than one reference. Graph genomes allow mapping of individual variation from single‐nucleotide polymorphisms through to major structural differences in the same species, which can comprehensively and accurately capture the differences between thousands of individuals (Rakocevic et al., 2019). This is particularly relevant for bivalves, as large‐scale structural variation between individuals and populations is almost certainly not being fully captured by the current generation of SNP level analyses (Calcino et al., 2021). However, while graph genomes are likely to become a feature of oyster genomics in coming years, this strategy will require a great deal of computing power. The benefits may only truly be realized when hundreds of individuals representing different populations have been sequenced, allowing population genetic parameters and structure to be more thoroughly characterized.

The next steps for oyster genome biology are likely to include the development of a phased (haplotype‐resolved) assembly of an individual (Cheng et al., 2022), capturing heterozygosity inclusive of structural differences and then most likely the development of a bivalve graph genome. Once in place, graph genomes will be added to and incrementally increase the accuracy of all genomic analyses. This includes transcriptomic analysis, studies of local genetic adaptation, genomic and epigenomic architectures of key traits, genome‐assisted breeding and possibly an understanding of the scope of the pan‐genome in bivalves. Most importantly, for the field to progress, the required collaborations must be forged from an early stage. Research communities for specific nonmodel organisms should coordinate and agree on a strategy for which specimens to sequence and why, then communicate openly and widely about this strategy. Here, we see not only the production of the first reference sequences for *O. edulis*, but also the beginnings of collaborations for the production of one of the first bivalve graph genomes.

## Data Availability

There is no data associated with this article.
